# Genome-Wide Identification, Expression Profile, and Alternative Splicing Analysis of the Brassinosteroid-Signaling Kinase (BSK) Family Genes in *Arabidopsis*

**DOI:** 10.3390/ijms20051138

**Published:** 2019-03-06

**Authors:** Zhiyong Li, Jinyu Shen, Jiansheng Liang

**Affiliations:** 1 Academy for Advanced Interdisciplinary Studies, Southern University of Science and Technology (SUSTech), Shenzhen 518055, China; 2 Department of Biology, Southern University of Science and Technology (SUSTech), Shenzhen 518055, China; 3 Co-Innovation Center for Modern Production Technology of Grain Crop, Yangzhou University, Yangzhou 225000, China; shenjinyu20160710@outlook.com

**Keywords:** brassinosteroid-signaling kinase, gene family, expression profile, alternative splicing, intron retention

## Abstract

Brassinosteroids (BRs) are steroid hormones essential for different biological processes, ranging from growth to environmental adaptation in plants. The plant brassinosteroid-signaling kinase (BSK) proteins belong to a family of receptor-like cytoplasmic kinases, which have been reported to play an important role in BR signal transduction. However, the knowledge of BSK genes in plants is still quite limited. In the present study, a total of 143 BSK proteins were identified by a genome-wide search in 17 plant species. A phylogenetic analysis showed that the BSK gene originated in embryophytes, with no BSK found in green algae, and these BSK genes were divided into six groups by comparison with orthologs/paralogs. A further study using comparative analyses of gene structure, expression patterns and alternative splicing of BSK genes in *Arabidopsis* revealed that all BSK proteins shared similar protein structure with some exception and post-translation modifications including sumolyation and ubiquitination. An expression profile analysis showed that most *Arabidopsis* BSK genes were constitutively expressed in different tissues; of these, several BSK genes were significantly expressed in response to some hormones or abiotic stresses. Furthermore, reverse transcription-polymerase chain reaction (RT-PCR) assays showed that *BSK5*, *BSK7*, and *BSK9* underwent alternative splicing in specific stress induced and tissue-dependent patterns. Collectively, these results lay the foundation for further functional analyses of these genes in plants.

## 1. Introduction

As sessile organisms, plants need to proceed in a coordinated manner to adapt to the constantly changing environment and react to stress conditions for growth and development. Phytohormones play a crucial role during these processes. Among them, brassinosteroids (BRs) are generally known as important plant hormones that play fundamental roles in various cellular, physiological, and developmental processes during plant life cycle [1,2]. 

To date, the BR signaling pathway has been well established and a number of the intracellular components of this pathway have been identified by genetic, genomic and proteomic studies. In the current model of the BR signaling pathway, the BR signal is perceived by BRASSINOSTEROID INSENSITIVE 1 (BRI1), a membrane-localized receptor kinase [3,4]. The direct binding of the BR ligand with BRI1 induces its association BRI1 with its co-receptor BRI1-ASSOCIATED RECEPTOR KINASE 1 (BAK1), enhancing the signaling output through reciprocal BRI1 transphosphorylation, inactivation of a glycogen synthase kinase-3 BRASSINOSTEROID INSENSITIVE 2 (BIN2) and activation of phosphatase BRI1 SUPPRESSOR 1 (BSU1), as well as the downstream transcription factors BRASSINAZOLE-RESISTANT 1 (BZR1) and BRI1-EMS-SUPPRESSOR 1 (BES1) [5,6,7,8]. Moreover, the transcription factor gene family, in turn, controls the expression of numerous target genes, which control various cellular, physiological, and developmental processes [6,9].

In the initial steps of BR signaling transduction, three receptor-like cytoplasmic kinases named BRASSINOSTEROID-SIGNALING KINASEs (BSKs) from *Arabidopsis* have been identified as BR-responsive proteins, including *BSK1*, *BSK2*, and *BSK3* [10]. *BSK1* and *BSK3* interact with BRI1 in vivo and are phosphorylated by BRI1 in vitro [10]. The phosphorylated BSK proteins further activate downstream phosphatase BSU1 for BR signaling transduction [6,11]. In *Arabidopsis*, there are 12 BSK proteins with putative kinase catalytic domain at the N-terminus and tetratricopeptide repeats (TPRs) at the C-terminus. However, the available results indicate that not all members are involved in BR signaling only. *BSK4*, *BSK6*, *BSK7*, and *BSK8* were reported to play a partial overlapping role in plant growth as well as in BR signaling with *BSK3* [12]. In contrast, *BSK3* was found as the only BSK member involved in BR-mediated plant root growth in a recent study [13]. Unexpectedly, the YODA mitogen-activated protein kinase pathway is activated by SHORT SUSPENSOR (SSP/BSK12) during embryogenesis, which has not been shown to be regulated by BRs [14]. In addition, the loss-of-function mutant *bsk5* is sensitive to salt stress and abscisic acid (ABA) hormone [15]. Silencing OsBSK1-2 inhibits flagellin- and chitin-triggered immune responses in rice [16]. Moreover, the *Arabidopsis* *BSK1* directly interacts with the immune receptor FLAGELLIN SENSING2 (FLS2) and further phosphorylates MAPKKK5 for the activation of pattern-triggered immunity (PTI) [17,18]. However, the detailed characterization of BSK family proteins and their functional importance in plants remains unclear.

In the present study, we screened the available genomes and identified a total of 143 BSK proteins from 17 plant species. We further performed a detailed analysis of their classification, phylogeny, and alternative splicing. Finally, we verified the expression profiles of the selected BSK genes in *Arabidopsis* by investigating their transcriptional levels upon exposure to abiotic stresses and hormones. Moreover, a novel post-transcription regulation pattern was found in several BSK genes, and potential significant functions of BSK genes were proposed. Our results provide important information about the evolution of the BSK gene family in plants and provide a basis for further studies of the functions of BSK family proteins.

## 2. Results

### 2.1. Identification and Characterization of the Brassinosteroid-Signaling Kinase (BSK) Genes in Plants

**Figure 1:**
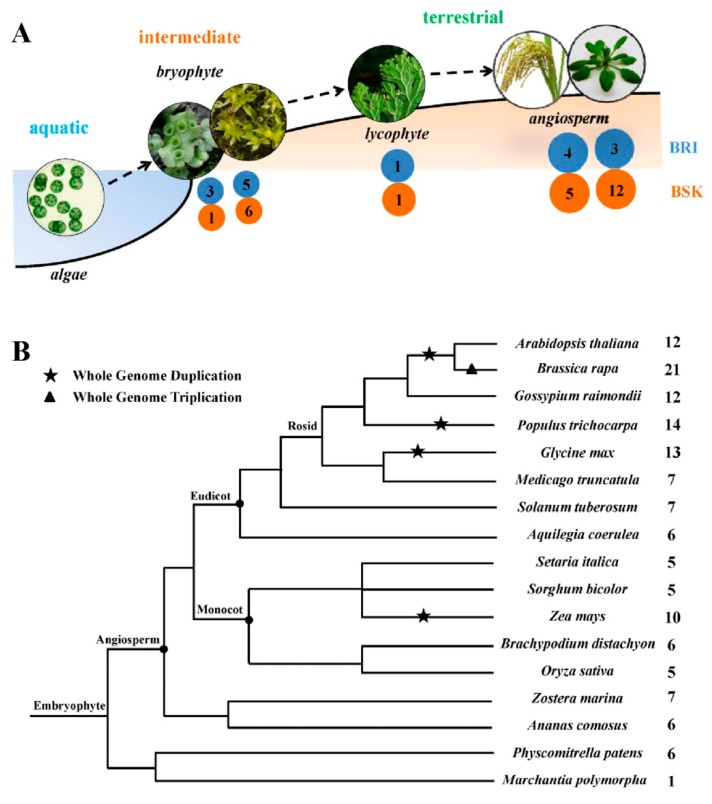
A comparative analysis of BSK genes in plants. (**A**) Evolution of core components of brassinosteroid (BR) signaling from aquatic plants to land plants indicated by dotted arrows. As representatives, component numbers of bryophyte, lycophyte and angiosperm were obtained from *Marchantia polymorpha*, *Physcomitrella patens*, *Selaginella moellendorffii*, *Arabidopsis thaliana*, and *Oryza sativa*, respectively. The numbers of BRI1-like and BSK proteins were indicated within blue and yellow circles, respectively. (**B**) The evolutionary relationships and the numerical details of the BSK family of each species.

In this study, a genome-wide analysis of the BSK gene family was performed on the basis of the completed genome sequences. Using the *Arabidopsis* Information Resource (TAIR), PlantGDB, Phytozome, and National Center for Biotechnology Information (NCBI) databases, we first retrieved the available BSK sequences from the currently sequenced genomes. A total of 17 plant genomes were analyzed to identify potential orthologous genes of BSK. These plants, representing the major clades of plants, included eight dicots (*Aquilegia coerulea*, *Arabidopsis thaliana*, *Brassica rapa*, *Glycine max*, *Gossypium raimondii*, *Medicago truncatula*, *Populus trichocarpa*, and *Solanum tuberosum*), five monocots (*Brachypodium distachyon*, *Oryza sativa*, *Setaria italica*, *Sorghum bicolor*, and *Zea mays*), and another four plant species (*Zostera marina*, *Ananas comosus*, *Physcomitrella patens*, and *Marchantia polymorpha*). The genome-wide analysis led to the identification of 143 BSK proteins in these plants. By investigating the extent of lineage-specific expansion of the BSK genes in plants, we found that the BSK gene originated in embryophytes (Figure 1A). Furthermore, almost all selected plants were found to have at least five BSK genes, with *B. rapa* having the highest number (21) of BSK genes (Figure 1B) and *M. polymorpha* having only one BSK gene. This result indicated that the BSK genes were subjected to a large-scale expansion in higher plants.

### 2.2. Phylogenetic Analysis of BSK Family Genes

**Figure 2:**
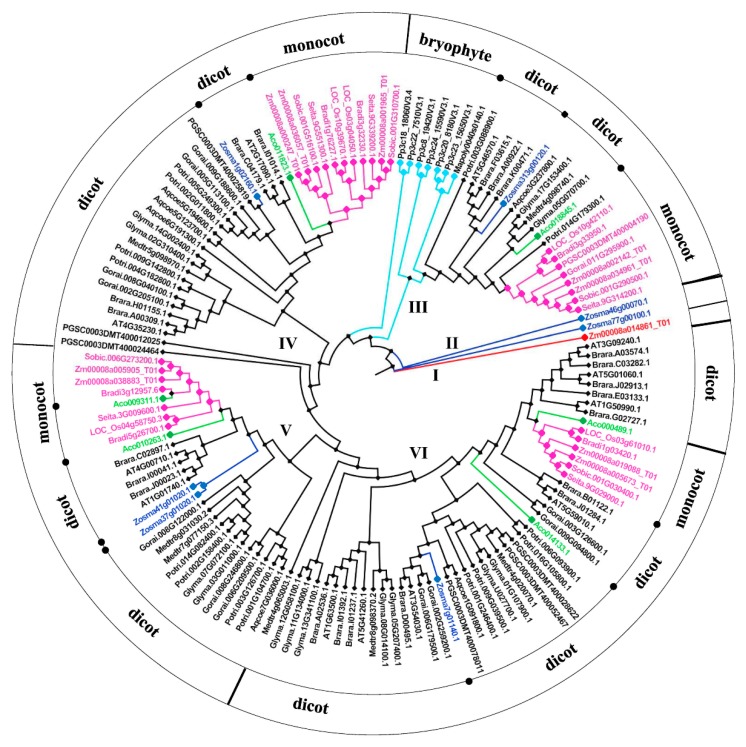
Neighbor-joining phylogenetic analysis of BSK genes. The gene tree was constructed using 143 BSK proteins and visualized using Figure Tree v1.4.2. Representative BSK proteins from different groups were marked with various colors. BSK proteins from dicot plants were in dark, and those from monocot were in pink. Blue and green ones were from *Zostera marina* and *Ananas comosus*, respectively.

To explore phylogenetic relationships among the 143 BSK proteins identified in different plant species, a neighbor-joining phylogenetic tree was constructed. This analysis divided all BSK proteins into six major groups according to the bootstrap values and phylogenetic topology (Figure 2). Only one protein from maize, named Zm00008a014861_T01, was in group I, which contained protein with longer amino acid sequence (743 aa) and an additional MSP (Major Sperm Protein) domain along with a conserved kinase domain and tetratricopeptide repeats (TPRs) (Figure S1). Group II was composed of two putative BSK proteins from the monocot seagrass *Z. marina*. The other four groups consisted of the BSK proteins largely from both dicot and monocot species. In particular, the BSK proteins from bryophytes including *P. patens* and *M. polymorpha,* were integrated into the group III. *A. comosus* and *Z. marina* belong to the angiosperm species prior to the split of eudicots and monocots. The phylogenetic analysis showed that the BSK proteins from *A. comosus* (Aco018845.1, Aco011823.1, Aco014133, Aco010223.1, and Aco000489.1) divided the BSK proteins from dicots and monocots in each group. Moreover, other five BSK proteins from *Z. marina* (Zosma313g00120, Zosma1g02160, Zosma37g01020, Zosma41g01020, and Zosma7g01140) further divided the BSK members from dicots into smaller groups. These results could be considered as evidence for lineage-specific expansion of the BSK genes after the divergence of dicots and monocots.

### 2.3. Genomic Structure and Conserved Motif Analysis of the BSK Gene Family

**Figure 3:**
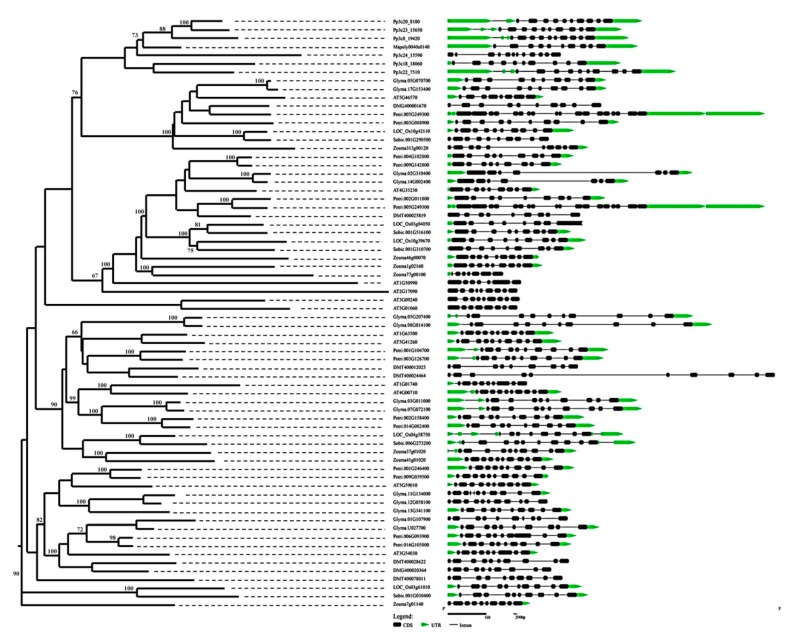
Phylogenetic relationship and exon-intron structure of BSK genes in representative plants. The BSKs were divided into five subgroups, which are indicated by different colors. The black boxes represent exons, solid lines represent introns, and green boxes represent untranslated regions (UTRs). The lengths of the BSK genes are indicated by horizontal lines (kb).

**Figure 4:**
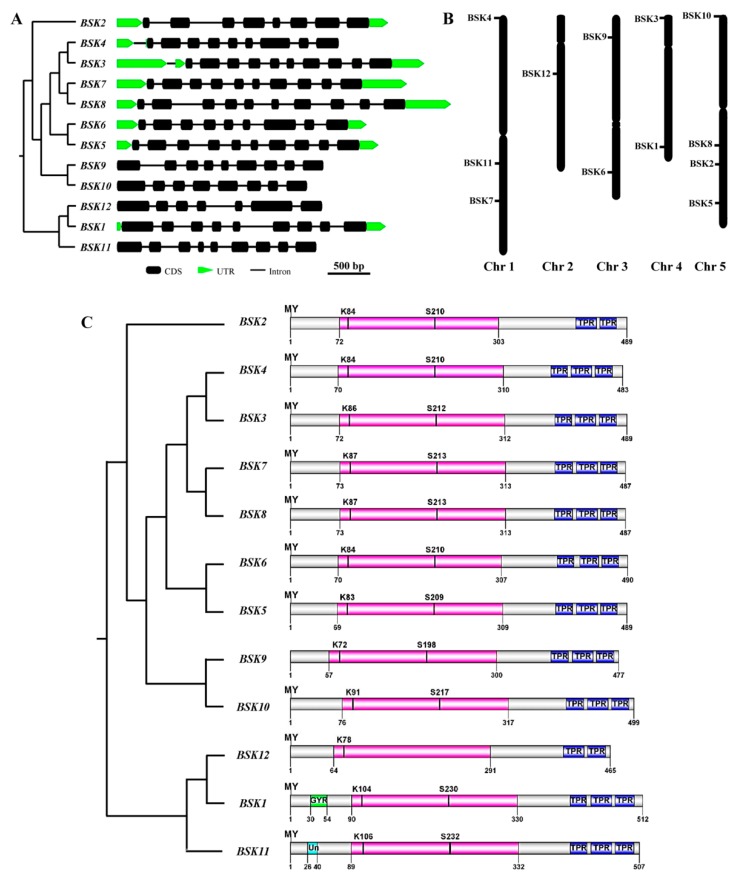
The features of the BSK genes in *Arabidopsis*. (**A**) Exon-intron structures of the BSK family genes. (**B**) Chromosomal location of BSK genes. (**C**) Schematic diagram of *Arabidopsis* BSK genes. The putative domains or motifs were identified using the Conserved Domain Database (CDD), Pfam and Simple Modular Architecture Research Tool (SMART) databases with the default parameters. The kinase domain was labeled in pink. MY, myrislation; TPR: tetratricopeptide repeat; GYR: glycine rich domain; Un: unknown domain.

The structure of the BSK genes was analyzed by comparing their full-length coding sequences (CDS) and the corresponding genomic DNA sequences using the software program GSDS 2.0 (http://gsds.cbi.pku.edu.cn/index.php). The exon/intron structure analysis showed that the structures of all BSK genes exhibited diversity both in their intron numbers and in their lengths (Figure 3). In particular, the intron numbers of the *Arabidopsis* BSK genes ranged between 6 and 9 (Figure 4A). Furthermore, we analyzed the different domain architectures, motif compositions, and gene structures of the BSK from *Arabidopsis*. The 12 BSK genes from *Arabidopsis* were located in all five chromosomes (Figure 4B). All BSK proteins had a putative kinase catalytic domain at the N-terminus and TPR repeats at the C-terminus, whereas *BSK2* and *BSK12* contained only two TPR repeats, one less than that from the other BSK members (Figure 4C). In addition, a glycine-rich region and unknown motif were found at the N-terminus in *BSK1* and *BSK11*, respectively. Previous studies showed that *BSK1* was phosphorylated by BRI1 at S-230, and a mutation in the ATP-binding site K-104 led to enzyme inactivity [10,17]. These conserved sites were also consistent with other BSK family members, except for *BSK12*. Moreover, most of the BSK proteins, except for *BSK9*, had a myristoylation site at the N-terminus (Figure S2). Other kinds of protein post-translational modifications, including sumoylation and ubiquitination, were also analyzed. It was found that all BSK family proteins possessed at least one sumoylation site and multiple ubiquitination sites, some of which were conserved (Figure S2). For instance, most of the ubiquitination sites were located in the kinase catalytic or TPR domain.

### 2.4. Expression Profiles Analysis of BSK Family Genes from Arabidopsis

**Figure 5:**
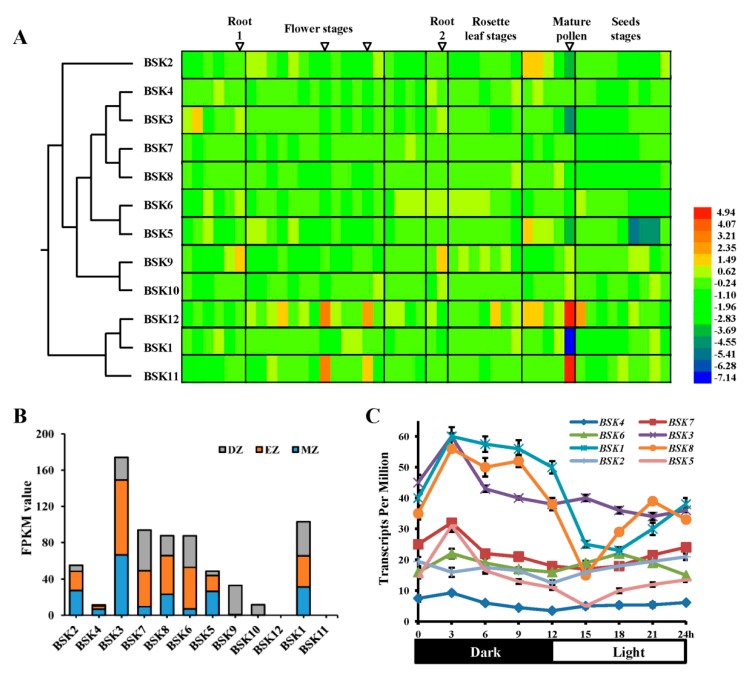
Development- and tissue-specific expression profiles of *Arabidopsis* BSK genes. (**A**) Heat map of the relative expression levels of all identified *Arabidopsis* BSK genes during development process. The colour bar represents the expression values. Samples from specific developing stages of *Arabidopsis* for gene expression analysis were highlighted with inverted triangles, such as roots from seedlings (1) and mature plants (2). (**B**) Expression profiles of BSK genes in specific root regions. DZ: division zone; EZ: elongation zone; MZ: mature zone. (**C**) Expression profiles of BSK genes across one light:dark cycle.

To compare the expression patterns of *Arabidopsis* BSK genes in different tissues or in responses to various abiotic stresses and hormones, the publicly available transcriptome sequencing and microarray datasets were analyzed. The results showed that most BSK members were constitutively expressed in different parts at different stages of plant development, while tissue-specific expression patterns were also observed (Figure 5A). For instance, *BSK1*, *BSK2*, *BSK3*, and *BSK5* were expressed at much lower levels in mature pollen, whereas *BSK11* and *BSK12* were more highly expressed. Among the 12 BSK genes, *BSK9* was specifically highly expressed in root, while *BSK5* was specifically expressed at very low levels during seed development. A cell-specific expression pattern analysis for the BSK family genes showed that most BSK family members possessed different expression levels within three root zones (Figure 5B). *BSK11* and *BSK12* were undetectable, whereas *BSK9* and *BSK10* were found only in the differentiation zones. Moreover, the latest RNA-seq transcriptome data analysis showed that the expression of some BSK family genes were light-dependent (Figure 5C). *BSK1*, *BSK3*, *BSK5,* and *BSK8* were found to be highly expressed at night, while their expression was significantly depressed upon exposure to light. By contrast, *BSK6* was expressed in a rhythmic manner independent of light.

**Figure 6:**
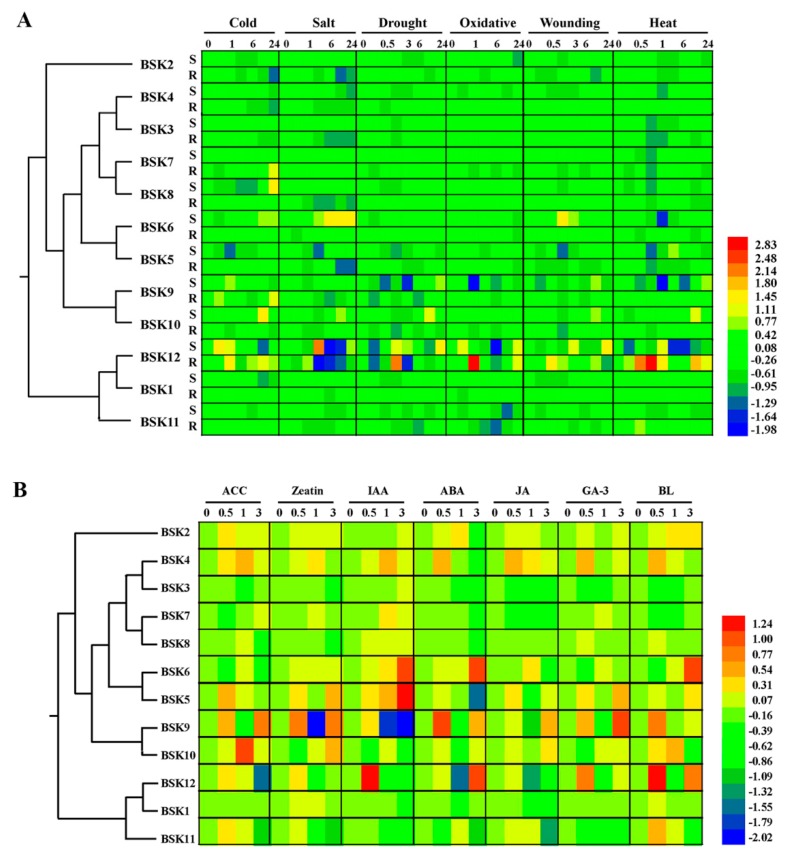
Expression patterns of all identified *Arabidopsis* BSK genes among hormone and abiotic stress. Public microarray data sets were obtained from BAR. Heat map representation the BSK genes under (**A**) six stress treatments, namely, cold, salt, drought, oxidative, wounding, and heat and (**B**) seven hormones, namely, ACC, zeatin, IAA, abscisic acid (ABA), methyl jasmonate (MeJA), gibberellic acid (GA-3), and brassinolide(BL). The colour bar represents the expression values.

Under various abiotic and biotic stress treatments, significant alterations in the transcript amounts of some BSK family members were observed at certain time points (Figure 6A). For instance, *BSK5* and *BSK9* showed significant responses to multiple stimuli, while at most times, these transcripts were down-regulated. *BSK6* was upregulated after one hour of exposure to salt or oxidative stress, whereas *BSK2* was strongly repressed after one hour of heat stress. Under phytohormone treatments, the transcript levels of *BSK5*, *BSK9,* and *BSK12* showed rapid responses with either up- or down-regulation (Figure 6B). Surprisingly, only *BSK12* was rapidly induced by BL, while the other three members (*BSK1*, *BSK2,* and *BSK3*) reported to be involved in BR signaling were insensitive to BL for three hours: this, however, remains to be further elucidated.

### 2.5. Diverse Gene Expression Patterns of BSK Family from Arabidopsis Using Reverse Transcription-Polymerase Chain Reaction (RT-PCR)

**Figure 7:**
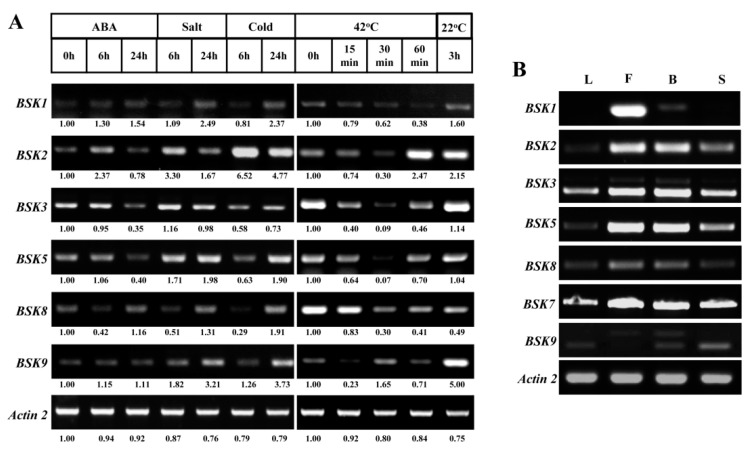
Reverse transcription-polymerase chain reaction (RT-PCR) validation of gene expression patterns of representative BSK genes under stresses and organs. Expression profiles of selected BSK genes under stresses (**A**) and in different tissues (**B**). For various stresses treatment, 7-day-old seedlings were subjected to 10 μM ABA, 150 mM NaCl and 4 °C on plates, respectively. Heat stress treatment was monitored with four-week-old seedlings under 42 °C for one hour and then recovered at 22 °C for three hours. The intensity corresponding to the individual PCR band was calculated by Image J with reactions from untreated samples as 1.00. L: leaves; F: flowers; B: buds; S: siliques.

The expression profiles of the representative BSK family members that demonstrated a diverse pattern of induced gene expression were validated by RT-PCR. The results showed that the expressions of some BSK genes were, indeed, regulated by stress or hormone treatments (Figure 7A). For instance, *BSK1* and *BSK3* were shown to significantly respond to salt, cold, and heat stresses after 6 or 24 h. In response to heat stress, *BSK2* was first sharply inhibited (within 30 min of the stress treatment) and then highly accumulated within one hour, and then kept constant after incubation at the normal growth condition for an additional three hours. However, it was also found that stress responses in some BSK genes were inconstant to the trends observed with the microarray data. For example, *BSK2* was found to be significantly depressed by cold and salt stresses in previous studies, but was observed to be strongly induced in our study. 

The tissue-specific expressional patterns of these BSK family genes were also investigated (Figure 7B). The results showed that promising expressional levels of *BSK2*, *BSK3*, *BSK5*, *BSK7*, and *BSK8* were found in leaves, flowers, fluorescents and siliques, which were consistent with the results from previous analysis. It also showed that all seven BSK genes were pronounced during reproductive growth. Our results further provided the information on the relative expression of these selected BSK genes. For instance, *BSK1* showed the strongest expression in flower and was dominantly expressed. Taken together, these results indicated the BSK genes are involved in multiple stress responses and developmental processes.

### 2.6. Alternative Splicing (AS) Analysis for BSK Family Genes in Arabidopsis

The precursor mRNAs (pre-mRNAs) with introns can be spliced by alternative splicing (AS) to generate multiple mRNAs that are translated into different proteins. By analyzing the genome annotation obtained from the Phytozome v12.1 database (https://phytozome.jgi.doe.gov), we found that many BSK genes from different species possessed several transcripts resulting from AS (Figure S3). In particular, the BSK genes from *P. patens* underwent extensive AS events, resulting in an increase in the complexity of the BSK gene family transcriptome (Figure S3K). On the contrary, only one BSK gene was found to exhibit AS in *Arabidopsis* and rice.

**Figure 8:**
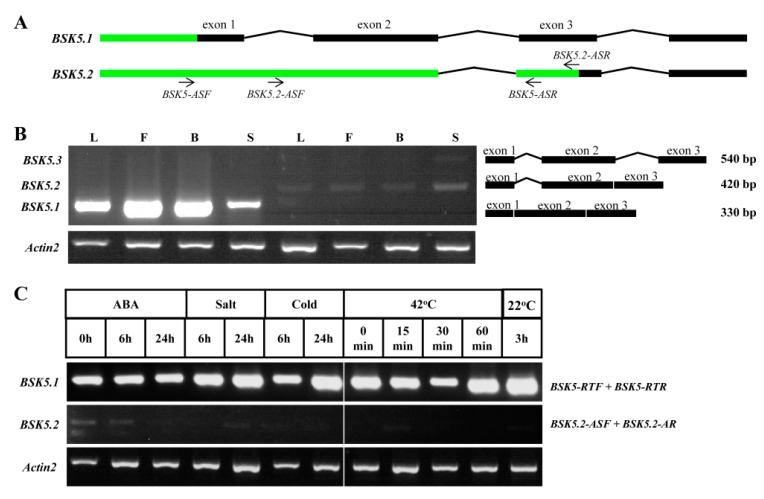
Alternative splicing analysis of *BSK5* in *Arabidopsis*. (**A**) The scheme shows the primers (black arrow) used in the PCR. The black boxes represent exons, solid lines represent introns, and green boxes represent untranslated regions (UTRs). BSK5-ASF, BSK5.3-ASF: forward primers; BSK5-ASR, BSK5.2-ASR: reverse primers. (**B**) The mRNA corresponding to the three variants of *BSK5* in different tissues was detected by RT-PCR. The numbers indicate the lengths (bp) of the spliced transcripts and schematic diagram outlining the organization of the transcript variants were on the right. L: leaves; F: flowers; B: buds; S: siliques. (**C**) RT-PCR analysis of the expression profiles of *BSK5* variants under stress treatment.

As annotated in the Phytozome database, *BSK5* had two spliced transcripts in *Arabidopsis* (Figure S3A). *BSK5.1* encodes a complete BSK protein, while the spliced transcript *BSK5.2* encodes an N-terminal-truncated BSK with the first intron retained from *BSK5.1*. To investigate the expression patterns of the two transcripts, specific primers were designed for RT-PCR (Figure 8A). Our results showed that *BSK5.1* was the dominant transcript, which was expressed in different tissues and responded to various environmental stimuli (Figure 8B,C). To our surprise, a third isoform *BSK5.3* was found in siliques (Figure 8B). A sequence analysis indicated two introns retained in *BSK5.3*, implying that *BSK5* spliced in a tissue-dependent manner. 

**Figure 9:**
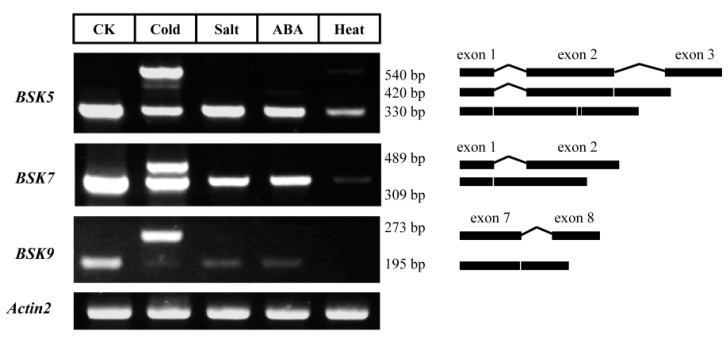
Intron retention analysis of BSK genes under stresses. Plants were stressed for 6 h under ABA/salt/cold and one hour under heat. Samples were then collected for mRNA isolation. Specific primers in exons were designed for RT-PCR in BSK genes. The detected PCR products were outlined on the right.

In addition to *BSK5*, we also detected a novel splicing transcript of *BSK9* in flowers and buds (Figure 7B). Further analysis showed that the intron between exons 7 and 8 was retained in *BSK9* (Figure 9). We further screened the ASIP (Alternative Splicing in Plants) database (http://www.plantgdb.org/ASIP/) for intron retention (IR) events among the BSK family genes in *Arabidopsis*, and there was only one record for *BSK7*. However, no IR event was detected in *BSK7* from the leaves under normal growth conditions (Figure 9). Instead, this IR event within *BSK7* was strongly induced by cold stress, and this same phenomenon was also found in *BSK5* and *BSK9*. Notably, *BSK5.3* was also found to be specifically induced by heat stress. These results indicated a novel post-transcriptional regulation pattern in the BSK genes.

## 3. Discussion

Several studies have reported the roles of BSK proteins from *Arabidopsis* and rice in BR signaling and immunity, as well as in abiotic stress responses [10,11,12,13,14,15,16,17,18,19]. However, the knowledge of BSK proteins in other plant species is still quite limited. In the present study, we identified a total of 143 BSK proteins from 17 plant species. Our analysis showed the evolutionary origin of BSK genes and BR receptor BRI1 or receptor-like genes in embryophytes, indicating the origin of BR signaling in plants (Figure 1A). A previous study reveals that plant BRI1 is highly conserved across taxa [20]. Taken together, the origin and development of the BR signaling system seems to be highly relevant with the evolution from aquatic to terrestrial plants, which have been observed in the ABA signaling system [21]. Bryophytes have an intermediate position between aquatic and terrestrial plants, and the establishment of the BR signaling pathway might have a great impact on the explanation of the movement of plants from water to land as an adaptation to environmental conditions. We also found evidence that genome duplication likely contributed the most to the expansion of the BSK gene family in many plant lineages. Prior to the split of eudicots and monocots, there was no evidence of whole-genome duplication (WGD). However, several rounds of WGD or triple genome duplication (WTD) have been reported in the 12 angiosperms studied here [22,23]. This might result in the expansion of the BSK genes from angiosperms. Indeed, we found that approximately two-fold expansion in the BSK genes was identified in the plants with WGD, including *A. thaliana*, *G.*, *P. trichocarpa*, and *Z. mays*. Furthermore, in *B. rapa*, we observed over three-fold BSK expansion, which was likely due to WTD.

In *Arabidopsis*, the BSK gene family includes 12 members, which share many similarities with each other with some exceptions. For instance, additional motifs were found in *BSK1* and *BSK11* (Figure 4C). The glycine-rich (GXXXG) motif, which provides conformational flexibility in the unstructured region, has been identified in many proteins, but has not yet been characterized in the BSK gene family [24]. It is thus, possible that this motif in *BSK1* might confer flexibility in the interaction of BSK with *BRI1* or in homo-dimmer conformation. Post-translational protein modifications, such as phosphorylation and myristoylation, have been reported in the BSK gene family. In the current study, no myristoylation site, which is critical for membrane association of BSK was found in *BSK9* [17]. A prediction of the subcellular localization of all BSK proteins from *Arabidopsis* showed that *BSK9*, as well as *BSK10* and *BSK11*, were absent from the plasma membrane, indicating that myristoylation might be sufficient, but not necessary, to anchor a protein to the membrane (Table S1). Further validation of the subcellular localization is required to support the accurate role of the myristoylation site. Other kinds of post-translational protein modification in this gene family were also investigated. The prediction results showed that conserved sumoylation and ubiquitination sites were found in all BSK family proteins (Figure S2). Overall, these post-translational protein modification sites were mainly located in the N- and C-terminals, where the residues were probably the targets of a SUMO or ubiquitin complex. Many proteins are both sumoylated and ubiquitinated, and often at the same lysine residues. In most BSK proteins, some conserved residues were found to serve as both ubiquitination and sumoylation sites. These two protein modifications can act either synergistically or antagonistically, indicating the existence of a fine-tuned post-translational regulatory mechanism of the BSK family, which remains to be elucidated.

In the present study, comprehensive microarray and RNA-seq analyses of the *Arabidopsis* BSK proteins at several developmental stages showed that some of these BSK proteins might extensively function in many tissues (Figure 5). Our RT-PCR data also showed that the selected BSK genes, including *BSK3*, *BSK7*, and *BSK8*, were expressed in leaves, buds, wide open flowers, and siliques; this result was consistent with the previous findings (Figure 7B). Some BSK proteins seemed to function in specific tissues, likely resulting in their sub-functionalization. For instance, *BSK12* was upregulated only at the early stages of see development, whereas the expression of *BSK5* was found to be reduced gradually; this was also verified by our RT-PCR data. BRs were reported to regulate seed size and shape in *Arabidopsis* by BZR1-mediated activation and repression of a number of known regulators of seed size [25]. AtBZR1-like genes are highly conserved in angiosperms, and both soybean GmBZL2 rice OsBZR1 play an important role in seed set/size [26,27,28,29]. Thus, it could be possible that *BSK5* and *BSK12* function upstream of BZR1 in seed development. BRs were also reported to play important roles in regulating root meristem maintenance and root elongation [30]. Here, significantly high expression of *BSK9* was observed in the meristematic zone of roots (Figure 6C). It could be exciting to examine the function of *BSK9* in root meristem development. Moreover, the expression of *BSK1* was the lowest in mature pollen among the BSK family genes (Figure 5A). On the contrary, *BSK11* and *BSK12*, which were in the same sub-group, showed much stronger expression. BRs have been reported to promote pollen germination and growth [31]. Some BSK downstream genes, including *Arabidopsis* BES1 and rice OsBZR1, have been found to be involved in anther and pollen development, indicating that these BSK members might play a role upstream of them in male gametophyte development [29,32]. In addition, BRs also regulate photomorphogenesis and link with light signals through BES1 [33,34]. Here, we found that several BSK family members showed light-dependent transcriptional regulation. For instance, the expressions of *BSK1*, *BSK3*, *BSK5*, and *BSK8* were significantly upregulated in the dark and repressed by light (Figure 5C). It will be interesting to investigate the function of these BSK members upstream of BZR1 in photomorphogenesis, broadening the functions of the BSK gene family.

A systematical microarray analysis of the BSK proteins under distinct abiotic stresses (cold, salt, drought, et al) and phytohormones (ABA, 1-aminocyclopropane-1-carboxylic acid (ACC), indole-3-acetic acid (IAA), BR, etc)) based on the *Arabidopsis* eFP Browser database indicated that most BSK proteins were involved in these abiotic stress and hormone responses (Figure 6). For instance, *BSK2*, *BSK3*, *BSK5*, *BSK6*, and *BSK12* were involved in the salt-stress response. Among these BSK genes, most were repressed by salt stress, whereas *BSK6* was constantly upregulated. In addition, *BSK5*, *BSK9,* and *BSK12* showed broad responses to multiple stresses. In contrast, only a small number of BSK genes were regulated by hormones. To our surprise, most BSK members, including *BSK1*, showed no response to BRs in 3 hours, whereas *BSK12* was sharply induced in 30 minutes, indicating an unexpected role of *BSK12* through BR perception (Figure 6B). Our RT-PCR results further confirmed that several selected BSK genes indeed showed a response to various abiotic stresses and hormones, although there was some inconsistency with the microarray data (Figure 7A). For instance, *BSK1* was upregulated under salt and cold stresses after 24 hours. Under the same situations, *BSK2* and *BSK9* were also highly induced. By contrast, all BSK genes were repressed by heat stress and ABA the inhibited expression of *BSK3*, *BSK5* and *BSK8*. Further experiments are required to elucidate the important roles of these BSK genes in various stress responses.

Alternative splicing, an important modulator of gene expression, can increase proteome diversity and regulate mRNA levels by generating multiple transcripts from the same gene [35,36]. In plants, this post-transcriptional mechanism is noticeably induced by environmental stimuli and plays an important role in the regulation of gene expression for biotic/abiotic stresse responses as well as for plant growth and development [37,38,39,40,41,42]. The RNA-seq analysis has revealed that in *Arabidopsis* more than 61% of intron-containing genes and even more undergo alternative splicing under normal growth and stress conditions, respectively [31]. Notably, IR is the most prevalent form of AS in higher plants. For instance, the AS forms of *Arabidopsis* contain an unusually high fraction of retained introns (above 30%), and more than half of the AS events belong to IR in rice [43,44,45]. IR is mostly accepted on account of mis-splicing and thought to be non-functional because they are likely to result in nonsense-mediated decay [46]. However, several studies have highlighted the functional importance of intron-retaining mRNAs in plants. IR in the *Arabidopsis* INDETERMINATE DOMAIN 14 (IDD14) transcription factor gene generates a competitive inhibitor that modulates starch accumulation in response to cold stress [47]. IR in the 5′ UTR of the Zinc-Induced Facilitator 2 gene (ZIF2) improves zinc tolerance in *Arabidopsis* [48]. *HAB1*, a group A protein type 2C phosphatase (PP2C), undergoes IR to produce a splice variant that plays opposing roles in fine-tuning ABA signaling [49]. Thus, IR should no longer be underestimated and exploring its roles in the development and/or stress response is of increasing importance. Here we found that the IR event in some *Arabidopsis* BSK genes was tissue-specific and responded to stress/hormone. The intron-containing form of *BSK9* preferred to express in development stages from buds to flowers, but was spliced completely in siliques. We also found that cold and heat stresses promoted the IR rate within the first two introns of *BSK5*, whereas ABA operated only in the first intron to generate and accumulate differently spliced transcripts. IR in *BSK7* and *BSK9* were also specifically induced by cold stress. Although the role of an individual transcript is largely unknown currently, these observations indicate the post-transcriptional regulation of these BSK genes in response to environmental stimuli. Future work will be performed to elucidate the mechanism and physiological significance of tissue-specific and stress-induced IR in these BSK genes.

Overall, our study represented diverse features of the BSK gene family in *Arabidopsis*, providing valuable insights into their important functions.

## 4. Materials and Methods 

### 4.1. Identification of BSK Family Genes and Phylogenetic Analysis of BSKs in Plants 

To identify ortholog(s) of AtBSK1 from *Arabidopsis* in the plant genome, the amino acid sequence of AtBSK1 was used as the seed sequence to perform a BLASTp search of the database whole genome sequences in the Phytozome database (https://phytozome.jgi.doe.gov). A similar method was applied on AtBRI1. Multiple protein sequence alignment for the deduced amino acid sequences of BSK proteins from different plant species was performed using the ClustalW software (http://www.clustal.org/clustal2/) with default settings. Then, the phylogenetic tree was constructed using MEGA software (Tokyo Metropolitan University, Tokyo, Japan) using the neighbor-joining (NJ) method and the bootstrap test carried out with 1000 replicates. The phylogenetic trees were visualized using FigTree (http://tree.bio.ed.ac.uk/software/figtree/).

### 4.2. Conserved Domain Recognition and Gene Structure Analysis

The CDS and the corresponding genomic sequences of the BSKs from the selected plant species were retrieved from the databases. Analysis of the exon-intron structures for the BSKs was carried out using the Gene Structure Display Server (GSDS v2.0, http://gsds.cbi.pku.edu.cn/index.php). Conserved domain for BSK proteins from *Arabidopsis* were identified using Conserved Domain Search Service (CD Search) from NCBI. The protein post-modification analysis was performed using SUMOgo (http://predictor.nchu.edu.tw/SUMOgo/) and UbiSite (http://csb.cse.yzu.edu.tw/UbiSite) with high specificity level of 95%. Subcellular prediction for BSK family genes were performed using Cell eFP Browser (http://bar.utoronto.ca/cell_efp/cgi-bin/cell_efp.cgi).

### 4.3. Expression Profiles Analysis Using Microarray and RNA-seq Data

For expression profiling of the BSK genes in *Arabidopsis*, we utilized the *Arabidopsis* ATH1 microarray data (*Arabidopsis* eFP Browser 2.0), root RNA-seq data bases (https://sites.lsa.umich.edu/pgrp-roots/) and AtRTD2 [50]. Different tissues of *Arabidopsis* and the seedlings under various stresses were analyzed. The gene expression patterns of each tissue were analyzed, and the expression values were log2 transformed from The Bio-Analytic Resource for Plant Biology (BAR). Finally, heat maps of hierarchical clustering were visualized using the Heml software. For data retrieved from RNA-seq data bases, the transcript abundance was expressed as fragments per kilobase of exon model per million mapped reads (FPKM) or Transcript Per Million.

### 4.4. Plant Materials, Growth Conditions and Stress Treatments

Seeds of *Arabidopsis thaliana* ecotype Columbia (Col-0) were used in this research. The seeds were surface sterilized with bleach and washed extensively with sterilized water for three times, and then incubated in a growth chamber after cold treatment for two days. After growth for one week, the seedlings were exposed to various stress conditions in plates containing 150 mM NaCl and 10 μM ABA, respectively. For cold and heat treatment, 7-day-old plants were placed at 4 °C or 42 °C incubator with normal illumination. Samples from leaves, buds, flowers and siliques were collected from well-growing plants at the same time. All the plant materials were harvested by liquid nitrogen cooling before stored at −80 °C.

### 4.5. RNA Extraction and RT-PCR

Isolation of total RNA from treated samples was performed using an RNA extraction kit (Promega, Madison, WI, USA). The cDNA synthesis was performed with total RNA (2 μg) reverse transcribed using All-In-One RT MasterMix (Applied Biological Materials, Zhenjiang, China). RT-PCR analysis was conducted using 2 × T5 Super PCR Mix (Tsingke, Beijing, China) and Taq Master MixTaq mix (Vazyme Biotech, Nanjing, China). All primers used in this study are listed in Supplementary Table S2. Quantification for gel intensity was carried out using Image J software (https://imagej.nih.gov/ij/).

## 5. Conclusions

In this study, we identified a total of 143 BSK proteins from 17 plant species. The phylogenetic analysis showed that the expansion of the BSK genes originated from embryophytes. A further comparative study revealed that most of the BSK genes in *Arabidopsis* were constitutively expressed and responded to some hormones or abiotic stresses. We also found some interesting post-transcriptional regulation patterns in *BSK5*, *BSK7*, and *BSK9*. Our results will further provide clues for the functional analysis of the important functions of BSK family genes in plants. 

## Supplementary Materials

The following are available online at https://www.mdpi.com/1422-0067/20/5/1138/s1.

## References

[1] Clouse S.D., Sasse J.M. (1998). BRASSINOSTEROIDS: Essential Regulators of Plant Growth and Development. Annu. Rev. Plant. Physiol. Plant. Mol. Biol..

[2] Fàbregas N., Caño-Delgado A.I. (2014). Turning on the microscope turret: A new view for the study of brassinosteroid signaling in plant development. Physiol Plant..

[3] Li J., Chory J. (1997). A putative leucine-rich repeat receptor kinase involved in brassinosteroid signal transduction. Cell.

[4] Wang Z.Y., Seto H., Fujioka S., Yoshida S., Chory J. (2001). BRI1 is a critical component of a plasma-membrane receptor for plant steroids. Nature.

[5] Li J., Wen J., Lease K.A., Doke J.T., Tax F.E., Walker J.C. (2002). BAK1, an Arabidopsis LRR receptor-like protein kinase, interacts with BRI1 and modulates brassinosteroid signaling. Cell.

[6] Kim T.W., Guan S., Sun Y., Deng Z., Tang W., Shang J.X., Sun Y., Burlingame A.L., Wang Z.Y. (2009). Brassinosteroid signal transduction from cell-surface receptor kinases to nuclear transcription factors. Nat. Cell Biol..

[7] He J.X., Gendron J.M., Yang Y., Li J., Wang Z.Y. (2002). The GSK3-like kinase BIN2 phosphorylates and destabilizes BZR1, a positive regulator of the brassinosteroid signaling pathway in *Arabidopsis*. Proc. Natl. Acad. Sci. USA.

[8] Yin Y., Wang Z.Y., Mora-Garcia S., Li J., Yoshida S., Asami T., Chory J. (2002). BES1 accumulates in the nucleus in response to brassinosteroids to regulate gene expression and promote stem elongation. Cell.

[9] Kim T.W., Wang Z.Y. (2010). Brassinosteroid signal transduction from receptor kinases to transcription factors. Annu. Rev. Plant. Biol..

[10] Tang W., Kim T.W., Oses-Prieto J.A., Sun Y., Deng Z., Zhu S., Wang R., Burlingame A.L., Wang Z.Y. (2008). BSKs mediate signal transduction from the receptor kinase BRI1 in Arabidopsis. Science.

[11] Mora-García S., Vert G., Yin Y., Caño-Delgado A., Cheong H., Chory J. (2004). Nuclear protein phosphatases with Kelch-repeat domains modulate the response to brassinosteroids in *Arabidopsis*. Genes Dev..

[12] Sreeramulu S., Mostizky Y., Sunitha S., Shani E., Nahum H., Salomon D., Hayun L.B., Gruetter C., Rauh D., Ori N. (2013). BSKs are partially redundant positive regulators of brassinosteroid signaling in Arabidopsis. Plant J..

[13] Ren H., Willige B.C., Jaillais Y., Geng S., Park M.Y., Gray W.M., Chory J. (2019). BRASSINOSTEROID-SIGNALING KINASE 3, a plasma membrane-associated scaffold protein involved in early brassinosteroid signaling. PLoS Genet..

[14] Bayer M., Nawy T., Giglione C., Galli M., Meinnel T., Lukowitz W. (2009). Paternal control of embryonic patterning in *Arabidopsis thaliana*. Science..

[15] Li Z.Y., Xu Z.S., He G.Y., Yang G.X., Chen M., Li L.C., Ma Y.Z. (2012). A mutation in Arabidopsis BSK5 encoding a brassinosteroid-signaling kinase protein affects responses to salinity and abscisic acid. Biochem. Biophys. Res. Commun..

[16] Wang J., Shi H., Zhou L., Peng C., Liu D., Zhou X., Wu W., Yin J., Qin H., Ma W. (2017). OsBSK1-2, an Orthologous of AtBSK1, Is Involved in Rice Immunity. Front Plant Sci..

[17] Shi H., Shen Q., Qi Y., Yan H., Nie H., Chen Y., Zhao T., Katagiri F., Tang D. (2013). BR-SIGNALING KINASE1 physically associates with FLAGELLIN SENSING2 and regulates plant innate immunity in Arabidopsis. Plant Cell.

[18] Yan H.J., Zhao Y.F., Shi H., Li J., Wang Y.C., Tang D.Z. (2018). BRASSINOSTEROID-SIGNALING KINASE1 Phosphorylates MAPKKK5 to Regulate Immunity in Arabidopsis. Plant Physiol..

[19] Yu M.-H., Zhao Z.-Z., He J.-X. (2018). Brassinosteroid Signaling in Plant–Microbe Interactions. Int. J. Mol. Sci..

[20] Navarro C., Moore J., Ott A., Baumert E., Mohan A., Gill K.S., Sandhu D. (2015). Evolutionary, Comparative and Functional Analyses of the Brassinosteroid Receptor Gene, BRI1, in Wheat and Its Relation to Other Plant Genomes. PLoS ONE.

[21] Umezawa T., Nakashima K., Miyakawa T., Kuromori T., Tanokura M., Shinozaki K., Yamaguchi-Shinozaki K. (2010). Molecular basis of the core regulatory network in ABA responses: Sensing, signaling and transport. Plant Cell Physiol..

[22] Panchy N., Lehti-Shiu M., Shiu S.H. (2016). Evolution of Gene Duplication in Plants. Plant Physiol..

[23] Del Pozo J.C., Ramirez-Parra E. (2015). Whole genome duplications in plants: An overview from Arabidopsis. J. Exp. Bot..

[24] Anand S., Sharma C. (2018). Glycine-rich loop encompassing active site at interface of hexameric, M. tuberculosis Eis protein contributes to its structural stability and activity. Int. J. Biol. Macromol..

[25] Jiang W.B., Huang H.Y., Hu Y.W., Zhu S.W., Wang Z.Y., Lin W.H. (2013). Brassinosteroid regulates seed size and shape in Arabidopsis. Plant Physiol..

[26] Zhang Y., Zhang Y.J., Yang B.J., Yu X.X., Wang D., Zu S.H., Xue H.W., Lin W.H. (2016). Functional characterization of GmBZL2 (AtBZR1 like gene) reveals the conserved BR signaling regulation in Glycine max. Sci. Rep..

[27] Manoli A., Trevisan S., Quaggiotti S., Varotto S. (2018). Identification and characterization of the BZR transcription factor family and its expression in response to abiotic stresses in *Zea mays* L.. Plant Growth Regul..

[28] Li Y.Y., He L.L., Li J., Chen J.G., Liu C.G. (2018). Genome-Wide Identification, Characterization, and Expression Profiling of the Legume BZR Transcription Factor Gene Family. Front. Plant Sci..

[29] Zhu X., Liang W., Cui X., Chen M., Yin C., Luo Z., Zhu J., Lucas W.J., Wang Z., Zhang D. (2015). Brassinosteroids promote development of rice pollen grains and seeds by triggering expression of Carbon Starved Anther, a MYB domain protein. Plant J..

[30] González-García M.P., Vilarrasa-Blasi J., Zhiponova M., Divol F., Mora-García S., Russinova E., Caño-Delgado A.I. (2011). Brassinosteroids control meristem size by promoting cell cycle progression in Arabidopsis roots. Development.

[31] Vogler F., Schmalzl C., Englhart M., Bircheneder M., Sprunck S. (2014). Brassinosteroids promote Arabidopsis pollen germination and growth. Plant Reprod..

[32] Ye Q., Zhu W., Li L., Zhang S., Yin Y., Ma H., Wang X. (2010). Brassinosteroids control male fertility by regulating the expression of key genes involved in Arabidopsis anther and pollen development. Proc. Natl. Acad. Sci. USA.

[33] Wang Z.Y., Bai M.Y., Oh E., Zhu J.Y. (2012). Brassinosteroid signaling network and regulation of photomorphogenesis. Annu. Rev. Genet..

[34] Wang W., Lu X., Li L., Lian H., Mao Z., Xu P., Guo T., Xu F., Du S., Cao X., Wang S. (2018). Photoexcited CRYPTOCHROME1 Interacts with Dephosphorylated BES1 to Regulate Brassinosteroid Signaling and Photomorphogenesis in Arabidopsis. Plant Cell.

[35] Mastrangelo A.M., Marone D., Laido G., De Leonardis A.M., De Vita P. (2012). Alternative splicing: Enhancing ability to cope with stress via transcriptome plasticity. Plant Sci..

[36] Marquez Y., Brown J.W., Simpson C., Barta A., Kalyna M. (2012). Transcriptome survey reveals increased complexity of the alternative splicing landscape in Arabidopsis. Genome Res..

[37] Staiger D., Brown J.W. (2013). Alternative splicing at the intersection of biological timing, development, and stress responses. Plant Cell.

[38] Ding F., Cui P., Wang Z.Y., Zhang S.D., Ali S., Xiong L.M. (2014). Genome-wide analysis of alternative splicing of pre-mRNA under salt stress in Arabidopsis. BMC Genomics.

[39] Feng J., Li J., Gao Z., Lu Y., Yu J., Zheng Q., Yan S., Zhang W., He H., Ma L., Zhu Z. (2015). SKIP confers osmotic tolerance during salt stress by controlling alternative gene splicing in Arabidopsis. Mol. Plant.

[40] Thatcher S.R., Danilevskaya O.N., Meng X., Beatty M., Zastrow-Hayes G., Harris C., Van Allen B., Habben J., Li B. (2016). Genome-Wide Analysis of Alternative Splicing during development and Drought Stress in Maize. Plant Physiol..

[41] Zhu F.Y., Chen M.X., Ye N.H., Shi L., Ma K.L., Yang J.F., Cao Y.Y., Zhang Y., Yoshida T., Fernie A.R. (2017). Proteogenomic analysis reveals alternative splicing and translation as part of the abscisic acid response in Arabidopsis seedlings. Plant J..

[42] Laloum T., Martín G., Duque P. (2018). Alternative Splicing Control of Abiotic Stress Responses. Trends Plant Sci..

[43] Ner-Gaon H., Halachmi R., Savaldi-Goldstein S., Rubin E., Ophir R., Fluhr R. (2004). Intron retention is a major phenomenon in alternative splicing in Arabidopsis. Plant J..

[44] Min X.J., Powell B., Braessler J., Meinken J., Yu F., Sablok G. (2015). Genome-wide cataloging and analysis of alternatively spliced genes in cereal crops. BMC Genomics.

[45] Wang B.B., Brendel V. (2006). Genomewide comparative analysis of alternative splicing in plants. Proc. Natl. Acad. Sci. USA.

[46] Lejeune F., Maquat L.E. (2005). Mechanistic links between nonsense-mediated mRNA decay and pre-mRNA splicing in mammalian cells. Curr. Opin. Cell Biol..

[47] Seo P.J., Kim M.J., Ryu J.Y., Jeong E.Y., Park C.M. (2011). Two splice variants of the IDD14 transcription factor competitively form nonfunctional heterodimers which may regulate starch metabolism. Nat. Commun..

[48] Remy E., Cabrito T.R., Batista R.A., Hussein M.A., Teixeira M.C., Athanasiadis A., Sá-Correia I., Duque P. (2014). Intron retention in the 5′UTR of the novel ZIF2 transporter enhances translation to promote zinc tolerance in Arabidopsis. PLoS Genet..

[49] Wang Z., Ji H., Yuan B., Wang S., Su C., Yao B., Zhao H., Li X. (2015). ABA signalling is fine-tuned by antagonistic HAB1 variants. Nat. Commun..

[50] Calixto C.P.G., Guo W.B., Jams A.B., Tzioutziou N.A., Entizne J.C., Panter P.E. (2018). Rapid and Dynamic Alternative Splicing Impacts the Arabidopsis Cold Response Transcriptome. Plant Cell.

