# Supplementary material for: Genome-Wide Identification, Expression Profile, and Alternative Splicing Analysis of the Brassinosteroid-Signaling Kinase (BSK) Family Genes in Arabidopsis
Source: Int J Mol Sci. 2019 Mar 6;20(5):1138. doi: 10.3390/ijms20051138 (PMC6429265; doi:10.3390/ijms20051138)
Supplement: Supplementary file 1 [file ijms-20-01138-s001.zip › ijms-457177 sp proof done/figure and table legends.pdf]

Figure S1. Protein structure prediction for Zm00008a014861_T01 using Conserved Domain Search Service (CD Search) from NCBI;

Figure S2. Putative conserved post-translation modifications in BSK family proteins from *Arabidopsis* predicted using SUMOgo and UbiSite. The kinase domain and TPR motif were labelled in pink and blue color, respectively. Two additional domains in BSK1 and BSK11 were marked in different colors;

Figure S3. Alternative splicing details and exon-intron structure of BSK genes annotated in all selected plant species: (A) *Arabidopsis thaliana*, (B) *Brassica rapa*, (C) *Glycine max*, (D) *Setaria italic*, (E) *Gossypium raimondii*, (F) *Populus trichocarpa*, (G) *Aquilegia coerulea*, (H) *Oryza sativa*, (I) *Brachypodium*, (J) *Medicago truncatula*, and (K) *Physcomitrella patens*. The black boxes represent exons, solid lines represent introns, and green boxes represent untranslated regions (UTRs);

Table S1. Subcellular localization prediction for *Arabidopsis* BSK family genes. Positive prediction was marked by tick;

Table S2. A list of primers used in this study.
