# Supplementary figures and images for: Genome-Wide Identification, Expression Profile, and Alternative Splicing Analysis of the Brassinosteroid-Signaling Kinase (BSK) Family Genes in Arabidopsis
Source: Int J Mol Sci. 2019 Mar 6;20(5):1138. doi: 10.3390/ijms20051138 (PMC6429265; doi:10.3390/ijms20051138)

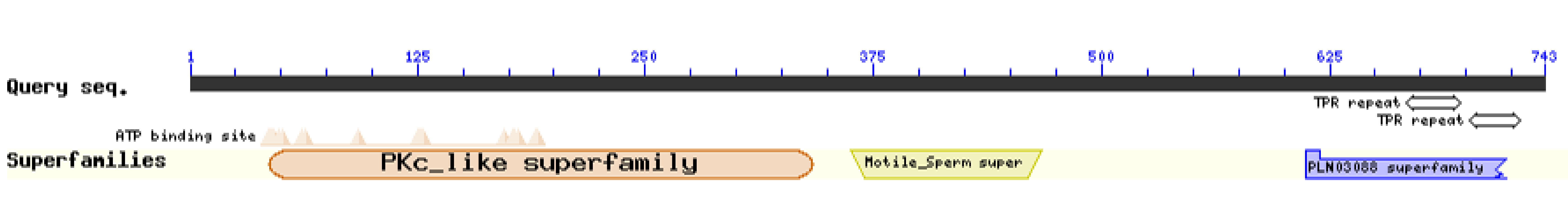

Supplement: Supplementary file 1 [file ijms-20-01138-s001.zip › ijms-457177 sp proof done/Figure S1.tif]

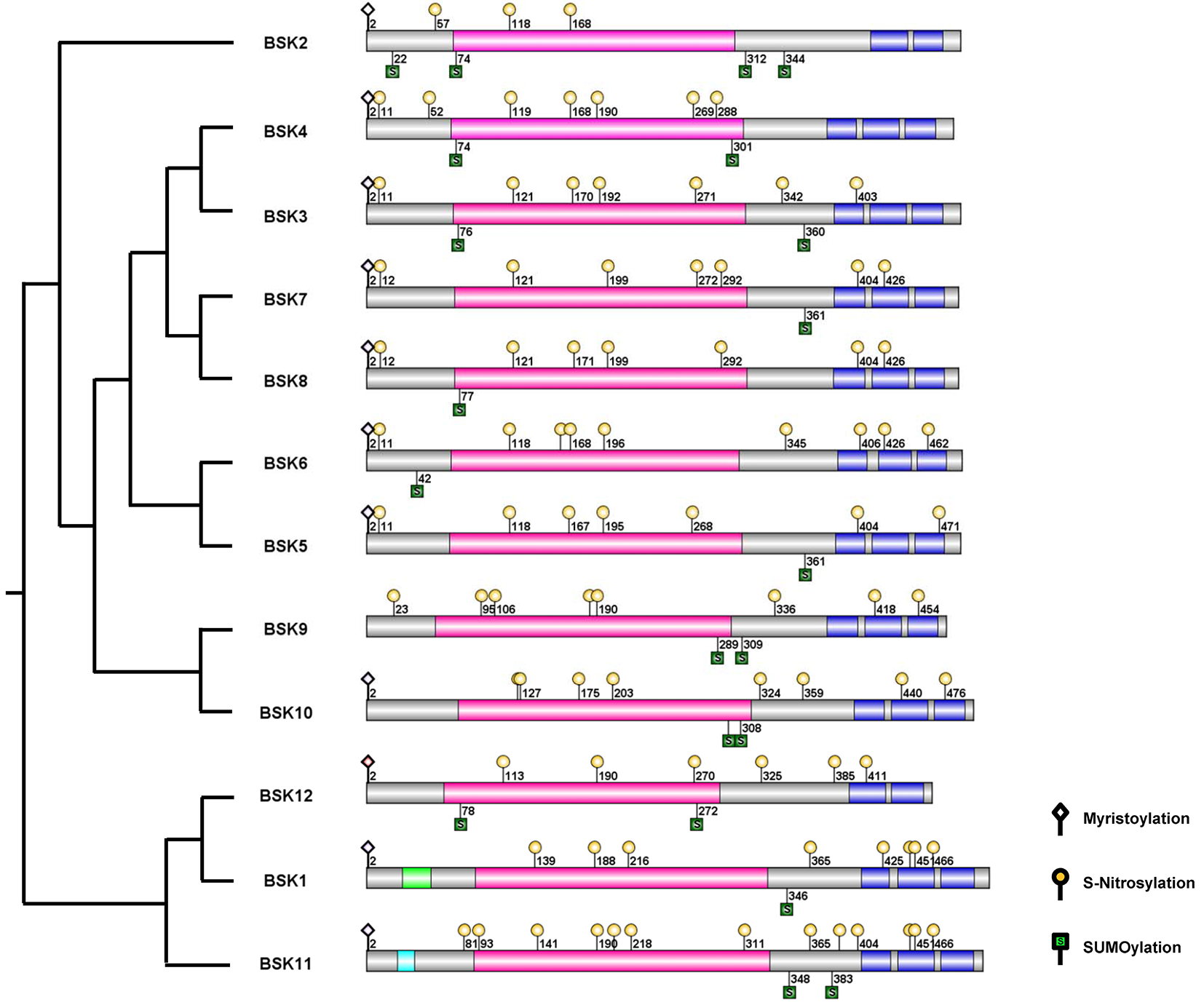

Supplement: Supplementary file 1 [file ijms-20-01138-s001.zip › ijms-457177 sp proof done/Figure S2.tif]

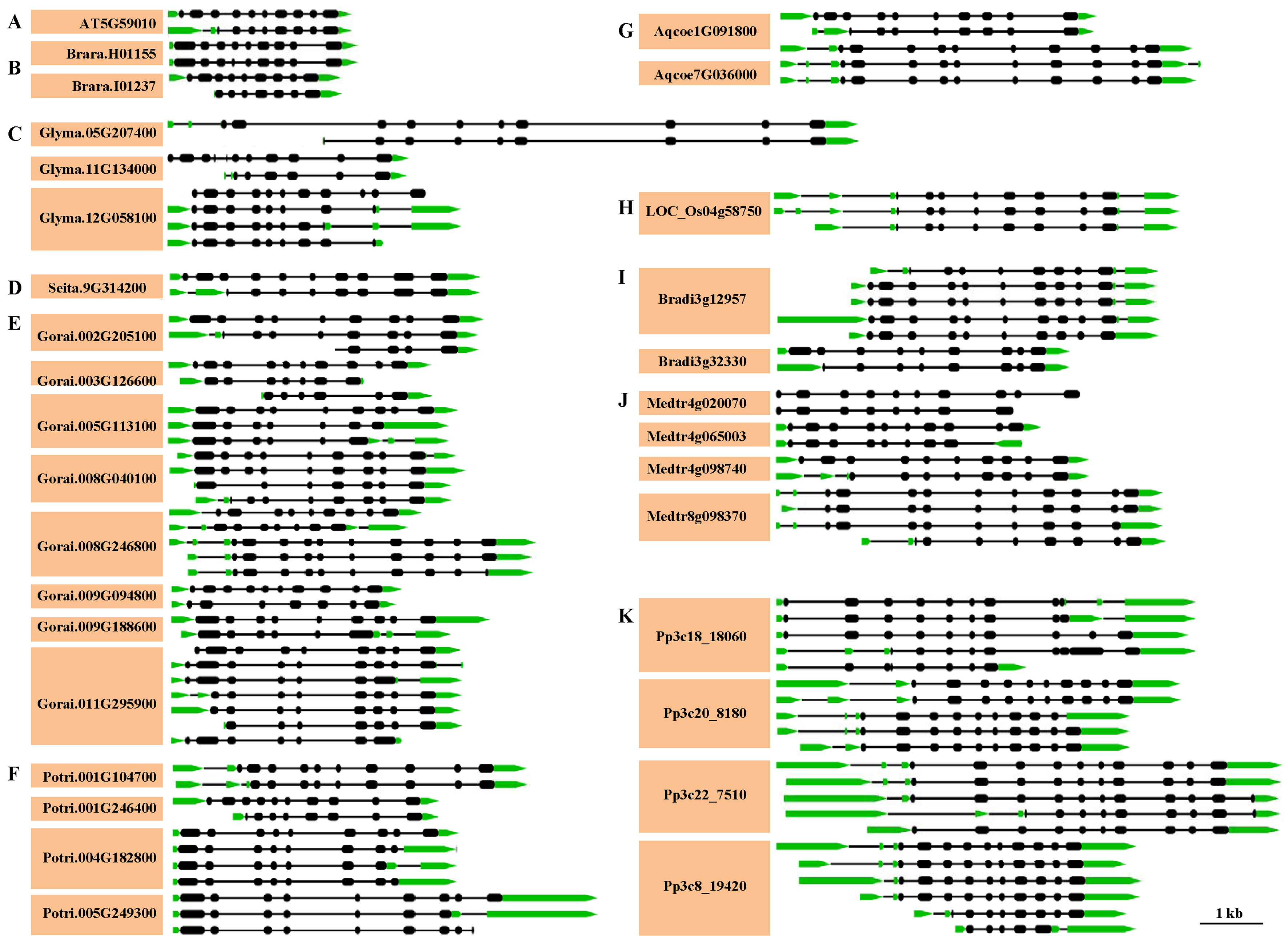

Supplement: Supplementary file 1 [file ijms-20-01138-s001.zip › ijms-457177 sp proof done/Figure S3.tif]
